# Doctors Dying

**Published:** 1870-12

**Authors:** 


### DOCTORS DYING.

The average life of physicians is less than that of the lawyer, and the clergyman by ten or fifteen years, from two main causes.

*First.* Irregular eating and sleeping.

*Second.* Increasing labor with increasing years. All other callings admit of agents and substitutes. Clerks and younger partners can do more of the routine and harder work in other callings, giving the head of the house more and more time for rest and quiet enjoyment. But no one can be the deputy of the old physician; he can have no substitute in the estimation of his patient, and each additional year increases the value of his advice; hence, the older he gets, the harder must he work, the less opportunity has he for repose, and he dies an age before his time.

Until forty, if the medical man lives that long—very many die even before that time—if he has a moderate constitution, he may be equal to the labors of his calling, but soon after, often ten years sooner than in ordinary callings, the head begins to whiten, the eyes to fail; weariness comes on earlier and more readily; the causes of disease meet with less and less resistance, vitality declines, and his work is done.

Medical men should early take note of these well ascertained facts, and begin at forty to enforce upon themselves the strict observance of those general laws of health whose importance they know so well, and not allow themselves to be so pressed by practice as to prevent the greatest abundance of regular sleep at night, the greatest regularity in eating, and the most prompt attention to the demands of nature. But, to be more concise still, not to go out until after breakfast, nor after ten o'clock at night.

